# Gender bias in hospitalization financing from borrowings, selling of assets, contribution from relatives or friends in India

**DOI:** 10.1016/j.socscimed.2020.113222

**Published:** 2020-09

**Authors:** Kaushalendra Kumar, Abhishek Singh, K.S. James, Lotus McDougal, Anita Raj

**Affiliations:** a International Institute for Population Sciences, Mumbai, India; b Center on Gender Equity and Health, Department of Medicine, University of California San Diego, San Diego, CA, USA; c Department of Education Studies, Division of Social Sciences, University of California San Diego, San Diego, CA, USA

**Keywords:** Hospitalization, Distressed hospitalization financing, Catastrophic hospitalization expenditure, Gender inequities, India

## Abstract

**Background:**

Studies from India have documented gender differentials in hospitalization financing. Much of this work focused either on children or adults, but not across age-groups. No research to date has focused on gender differentials in case of catastrophic hospitalization expenditures. This study assesses gender differentials in distressed financing (borrowing, selling of assets, contributions from relatives or friends) for hospitalization in cases of catastrophic expenditures for hospitalization in India, for young, adult and older adult patients.

**Methods:**

We conducted a cross-sectional analysis of India’s 2017-18 National Sample Survey, which collected data on hospitalization and expenditures. We used multivariable probit regression and adjusted marginal effects to assess the associations between gender and the use of distressed financing for catastrophic hospitalization expenditures. Models were stratified by age, and run both with and without sample selection. Secondary analyses assessed gender differentials in the use of distressed financing for hospitalization in case of health insurance cover or not.

**Results:**

Multivariable sample selection-adjusted probit regression shows that in households which incurred severe catastrophic hospitalization expenditures, the probability of using distressed financing for hospitalization of young or older females was 10% points lower than their male counterparts. In households which did not incur severe catastrophic hospitalization expenditures, there was no significant gender differential in use of distressed financing for hospitalization for any age group. In households which incurred severe catastrophic hospitalization expenditures, the probability of using distressed financing for hospitalization was lower for older females than for older males irrespective of health insurance cover.

**Conclusion:**

There appears to be a clear gender discrimination in distressed financing of hospitalization costs among younger and older individuals in households that incurred severe catastrophic hospitalization expenditures in India. Health systems should consider how to otherwise support necessary hospitalization financing for girls and older women.

## Highlights

• Young and older females experience bias in hospitalization financing.
• Biases were concentrated in severe catastrophic hospitalization expenditures.
• Health insurance did not protect older females against this gender bias.

## 1 Introduction

While access to hospitals has improved over the past two decades in India, utilization has also increased, with hospitalization rates more than doubling between 1995 and 2014 (GoI, 2018; Pandey et al., 2017). This surge in hospitalization rates has been driven by a four-fold increase in hospitalizations due to non-communicable diseases (NCDs), compared with a two-fold increase in hospitalization due to communicable diseases (CDs), and is particularly prominent for infants and older adults relative to those of childbearing age (Kastor and Mohanty, 2018a; Pandey et al., 2017b). Unfortunately, the costs of hospitalization have also increased by 79% in this same period (Kastor and Mohanty, 2018b). Non-communicable diseases, the most common reason for hospitalizations among older adults, are more than twice as costly to treat as communicable diseases, which are the most common cause of hospitalizations for infants (Kastor and Mohanty, 2018a). As a majority (63%) of health expenditures in India are paid with out-of-pocket expenditure (NHSRC, 2019), households first rely on readily available funds such as income or savings. However, in cases of catastrophic health expenditures, households turn to resources like borrowing, selling of assets, and/or contribution from relatives or friends, henceforth referred to as distressed financing (Joe, 2015; Kruk et al., 2009; Leive; Xu, 2008). But, health insurance protects the household from incurring high out-of-pocket expenditure and incurring catastrophic health expenditure (Kastor and Mohanty, 2018a; Kruk et al., 2009; Kumar et al., 2015).

Theoretically, the household as a unit of production generates utilities or welfare from health investments in its members, which can be aggregated at the household level (Becker, 1965; Haddad et al., 1998; Samuelson, 1956). Households allocate limited financial resources for health care financing either to equalize the utilities of each household member or to efficiently maximize the total household utility (Becker, 1974; Haddad et al., 1998; Joe et al., 2017). Household utility maximization depends first on household members’ endowment – wealth, regular salary/wage/pension earning, and education; and second on bargaining power which emanates from gender, relationship to household head, and marital status (David and Kaplan, 1995; Joe et al., 2017). For a given bargaining power, there is less endowment among young and older adult females compared with males in India (Ghani et al., 2012; Sengupta, 2016). Hence, as households consider how to prioritize expenditures for health financing, too often lesser valuation of women and girls in family may affect decision-making, resulting in unequal access to necessary health care services (IIPS and ICF, 2017; Joe, 2015; Joe et al., 2017; Mathew, 2012; Vilms et al., 2017).

Research from various developed and developing countries have shown mixed results when it comes to gender differentials in health seeking and health expenditure. Studies from South Korea and China suggest that men are more likely than women to use health care services, and to spend more on those services (Ahmed et al., 2003; Song and Bian, 2014). In contrast, a study from Vietnam did not show any gender differential in health care utilization (Pham et al., 2018). Moreover, studies from some higher income countries have shown higher health care utilization among adult and older adult females compared to their male counterparts (Gajovic et al., 2019; Roy and Chaudhuri, 2012; Verbrugge et al., 1987). A pooled analysis of emerging economies – China India, Mexico, Ghana, the Russian Federation, and South Africa – revealed that women older than 50 years were less likely to use inpatient care and more likely to use outpatient care compared with male counterparts Peltzer et al. (2014).

Studies among infants and young children in India indicate that parents are less likely to seek health care for girls compared to boys (IIPS and ICF, 2017; Mathew, 2012; Vilms et al., 2017). Further, when families seek care, higher expense private care is more likely to be used for boys than girls (Willis et al., 2009). While this bias appears to be greater for older relative to early adolescent children, healthcare use is more likely to be sought for boys even among youth aged 10–14 (Gupta et al., 2016). There is some evidence of gender bias in care seeking for adults, as well, with indication that this bias may be even stronger for adults, particularly older adults, relative to children in India (Moradhvaj and Saikia, 2019; Pandey et al., 2017). While adult and older adult women suffer from higher rates of chronic disease and other morbidities relative to male counterparts (NSSO, 2019; Patel et al., 2019; Saikia et al., 2016), they are less likely than men to be hospitalized or receive medical care when needed (Joe et al., 2017; Moradhvaj; Saikia, 2019). These findings suggest that in a context of rising medical costs and absence of public health care provisions or health insurance cover, households may opt to forgo care or reduce health expenditures for female but not male household members.

Though some research exists on gender differentials in treatment seeking, hospitalization, and health expenditures in India (Gupta et al., 2016; IIPS and ICF, 2017; Joe, 2015; Joe et al., 2017; Pandey et al., 2017a; Saikia et al., 2016; Willis et al., 2009), only four studies have explored the gender differentials in hospitalization financing strategies (Asfaw et al., 2010; Joe, 2015; Joe et al., 2017; Moradhvaj and Saikia, 2019). National data from India demonstrate greater use of distressed financing for hospitalization (DFH) of adult men than adult women (Moradhvaj and Saikia, 2019), for children under 10 (Asfaw et al., 2010), and older adult (Joe et al., 2017). Joe (2015) has shown that irrespective of age, DFH was higher among males compared with females. These findings suggest gender bias in the use of distressed financing to pay for hospitalizations costs, but have not examined such biases through two key lenses.

**Fig. 1:**
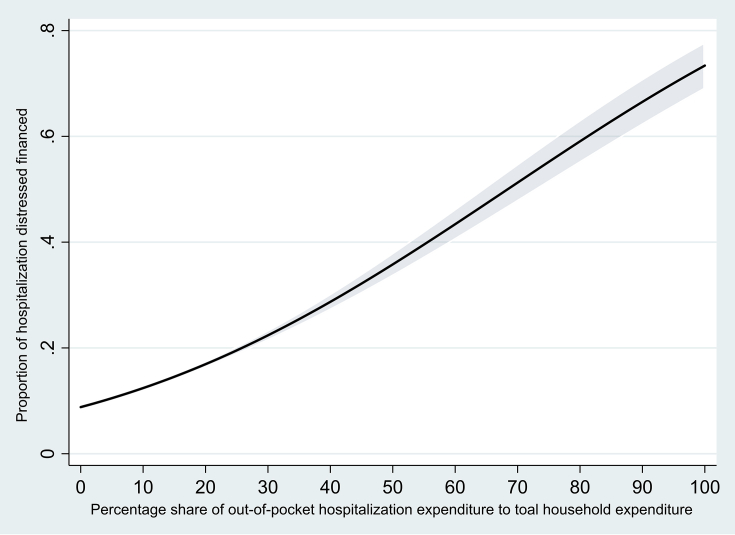
Proportion hospitalization cases distressed financed by percentage share of out-of-pocket hospitalization expenditures to total household expenditures, India 2017-18.

First, prior research has not differentiated between non-catastrophic and catastrophic hospitalization expenditures in India; we believe that households incurring catastrophic health expenditures are more likely to resort to borrowings/selling of assets/contribution from friends or relatives for hospitalization financing (Kastor and Mohanty, 2018a). Indeed, it is clear that the use of distressed financing increases with increases in share of out-of-pocket health expenditures to total household expenditures (See Fig. 1).

Further, no analyses have considered these issues across the life course, comparing gender bias in hospitalization financing for the young, adults with earning potential, and older adults, ignoring the differential social and economic roles played by women of different ages. For example, adult women may contribute to childcare, household labour, and earnings more than those older or younger women, and may thus receive greater priority in the use of DFH. In comparison, older adult women, due to their lower economic value in the household, may receive lower priority (Craigie and Dasgupta, 2017; Sengupta, 2016). Additionally, in patriarchal societies such as India, parental investment on female children accrue dividend to their in-laws after their marriage. Hence, household may discriminate against young female in DFH. Finally, previous studies have also not examined gender bias in use of DFH in case of intersection of age groups, catastrophic hospitalization spending, and health insurance cover.

The goal of this paper is to address this gap in knowledge through an in-depth examination of gender differentials in the use of DFH in cases of catastrophic expenditure across different age groups. Specifically, we examine gender differentials in DFH in households that incurred catastrophic hospitalization expenditures for young (aged 0–14 years), adult (aged 15–59 years), and older adult (aged 60+ years) patients. We additionally examine gender differentials in use of DFH in case of health insurance cover or not.

## 2 Data and method

### 2.1 Data

Data were taken from 75th round of the Social Consumption in Health Survey, a nationally and state/Union Territory representative sample of households in India assessing socioeconomic and demographic factors, general consumer expenditure, and hospitalization (in-patient treatment) in the past 365 days. For each hospitalization, the survey assesses a) nature of ailment, b) nature of treatment, c) level of care, d) duration of treatment (in days), e) total expenditure for that hospitalization (including consultation, medication, transportation), and f) sources of financing for the hospitalization expenses. Data were collected from July 2017 to June 2018 by the National Sample Survey Office (NSSO), Ministry of Statistics and Programme Implementation, Government of India (NSSO, 2019). Adopting a multi-stage stratified sampling method, 557,889 individuals (were recruited from 113,823 households across 14,258 rural villages/urban blocks in India. Additional survey details are available elsewhere (NSSO, 2019).

### 2.2 Analytic sample

The analytic sample is comprised of the subsample of hospitalized cases and reported catastrophic expenditures due to hospitalization in the past 365 days (n = 93,925, excluding 6 individuals who self-identified as transgender).

Catastrophic health expenditures occur when share of out-of-pocket health spending crosses a certain threshold of total household consumption expenditure or household's capacity to pay (Kumar et al., 2015; Wagstaff; Doorslaer, 2003; Wagstaff et al., 2018; Xu et al., 2003). While there is no consensus on total household consumption or household's capacity to pay to be used to define catastrophic health spending and the threshold limit, we use total household expenditure and follow thresholds used in multiple studies and the Sustainable Development Goals, namely 10% and 25% cut points (Pandey et al., 2018; Wagstaff et al., 2018; WHO, 2020). In this context, we have thus defined hospitalization expenditures as: no catastrophic hospitalization expenditures (out-of-pocket hospitalization expenditures comprise under 10% of total household expenditures), moderate catastrophic hospitalization expenditures (out-of-pocket hospitalization expenditures comprise 10% or more of total household expenditures) and severe catastrophic hospitalization expenditures (out of pocket hospitalization expenditures comprise 25% or more of total household expenditures). Henceforth, these three hospitalization expenditures will be referred to as NCHE, MCHE, and SCHE, etc.

The two main components used to calculate whether or not hospitalization expenditures were catastrophic are out-of-pocket hospitalization expenditure (OOPHE) and total household expenditure (THE). OOPHE is the net of reimbursement, total expenditure for treatment during hospitalization on doctor's/surgeon's fee (hospital staff/other specialists), medicines, diagnostic tests, bed charges, other medical expenses (attendant charges, physiotherapy, personal medical appliances, blood, oxygen, etc.), transport for patient, other non-medical expenses incurred by the household (food, transport for others, expenditure on escort, lodging charges if any, etc.), and medical insurance premium. Total household expenditure includes - 1) household's usual consumer expenditure (comprised of usual expenditure for household purposes, expenditure on purchase value of any household durables, and market value of consumption from wages in kind, home-grown stock, and free collection), 2) out-of-pocket hospitalization expenditure, and 3) medical insurance premium. Notice that estimate of OOPHE depends on recall or reference period of the survey, number of items related to hospitalization expenditure collected, and wording of questions (Kumar et al., 2015; Raban et al., 2013; WHO, 2011). However, studies have shown that OOPHE computed using the disaggregated information on expenditure for each episode of hospitalization in last 365 days in NSSO are reasonably reliable (Pandey et al., 2018; Raban et al., 2013).

In order to make financing assessment comparable by sex, we excluded hospitalized female cases specific to maternal health, including pregnancy-related complications, childbirth, and post-partum maternal complications, resulting in a subsample of N = 64,799 hospitalized cases. Hence, the final analytic sample includes 27,323 hospitalization cases of MCHE (n = 3484 young hospitalized cases, n = 16,758 adult hospitalized cases, and n = 7081 older adult hospitalized cases) and 11,908 hospitalization cases of SCHE (n = 1225 young hospitalized cases, n = 7254 adult hospitalized cases, and n = 3429 older adult hospitalized cases).

### 2.3 Measures

The dependent variable is the use of distressed financing for hospitalization. For each hospitalization, NSSO asked the respondents about the major source of finance for expenses, across the following response categories: income/savings, borrowing, sale of physical assets, contributions from friends and relatives, and other sources. Major sources of finance were reported for almost all (99.7%) hospitalizations, while only one third (33.3%) of the hospitalized cases reported a secondary important source of finance for expenses. We thus defined the use of DFH as a binary variable identifying whether the major source of finance for hospitalization expenses came from borrowings, sale of assets, and contributions from friends/family (yes), or none of those sources (no).

**The independent variable** is sex (male/female) of the hospitalized cases.

**Control variables** focus on hospitalization & treatment characteristics, economic characteristics, and social characteristics. Hospitalization and treatment characteristics include number of hospitalizations in household, nature of ailment, whether hospitalization was at a private health care facility, duration of hospitalization (days), out-of-pocket hospitalization expenditure (OOPHE), and share of OOPHE to total household non-medical consumption expenditure. The number of household hospitalizations over the past 365 days, across all household members, is included to account for increased economic costs for the household as a whole associated with more hospitalizations. Since hospitalizations with certain diseases may lead to higher expenditure, we classified the nature of ailment for the index hospitalization into five categories: cardio-vascular diseases (CVD), cancers, musculoskeletal diseases, communicable diseases, and other diseases (See Appendix Table 1). As per the NSSO (2019), average expenditure for hospitalization was highest for cancers followed by CVD and musculoskeletal diseases. On the other hand, communicable diseases were more common among the young. Moreover, studies have shown that households seeking treatment for CVD and cancers are more likely to borrow or seek contribution from friends/relatives (Joe, 2015).

We classified hospitalization in private hospital as private health care and inpatient treatment in government/public hospital and charitable/trust/NGO run hospital were classified as not receiving private care. Duration of hospitalization was assessed as number of days (continuous) that the index case remained hospitalized for a treatment in last 365 days. Duration of hospitalization is associated with nature and severity of ailment, as well as the treatment and level of care required (Kastor and Mohanty, 2018a). Since the OOPHE may vary by the nature of ailment, and certain ailments may be higher among one gender than another, we also included natural log of OOPHE in the statistical models. We included share of OOPHE to total household expenditure in the regression models as it measures the economic burden of hospitalization.

Economic characteristics include non-medical monthly per capita consumption expenditure, health insurance cover, regular salary/wage employee, and currently pensioner/rentier/remittances recipient. Non-medical monthly per capita consumption expenditure (MPCE), which measures the household economic status (Srivastava and Mohanty, 2010), is calculated after dividing the household's reported usual monthly consumer expenditure (in Indian Rupees [₹]) by the number of household members. Health insurance in our analysis includes any scheme - government sponsored (e.g. RSBY, Arogyasri, etc.), government/PSU as an employer (e.g. CGHS, reimbursement from govt. etc.), employer supported (other than government/PSU) health protection (e.g. ESIS), arranged by household with insurance companies, and others. Income and savings can be used to pay for any contingent and involuntary health expenditures. In the present study, income has been measured by usual principal activity status (UPAS) of household head for young and adult and index hospitalized case's UPAS for adult and older adult. Two categories of UPAS – 1) worked as regular and salaried wage employee and 2) pensioner/rentier/remittances recipients categorized as yes or no.

Other control variables include individual and household characteristics - secondary education completed, currently married, female household head, scheduled caste/tribe, Hindu, rural residence. While for young hospitalization, we assessed education for head of household, index case education is assessed for adult and older adult hospitalization. Caste was categorized into Scheduled castes/tribes or others. Likewise, religion was categorized as Hindu or others. For adults and older adults, marital status was categorized into currently married or others. For the young and adult participants, we additionally assessed whether the head of the household was a female.

### 2.4 Analysis

Our primary analysis used bivariate log probability models (McCullagh and Nelder, 1989) to assess bivariate associations between gender and use of DFH, as well as between use of DFH and other control variables. We used multivariable probit models (Chib and Greenberg, 1998) for estimating adjusted marginal effects of gender on use of DFH. All regression models were stratified analyses by age, categorized as 0–14 (young), 15–59 (adult, working age), and older adult (60+).

There is a substantial risk of selection bias in these data, as information on the cause of hospitalization, expenditures, and sources of financing is collected from only hospitalized cases. Previous studies have assumed that household decisions regarding hospitalization and the financing thereof occur contemporaneously (Asfaw et al., 2010; Moradhvaj and Saikia, 2019). However, gender bias starts from the occurrence of disease, as household first need to consider whether sick individuals should indeed be hospitalized (IIPS and ICF, 2017; Mukherjee and Karmakar, 2008; Saikia et al., 2016; Vilms et al., 2017; Willis et al., 2009), and then decide on how to finance that hospitalization. Sources of hospitalization financing is contingent upon household's decision to seek treatment which itself depends upon demographic, socioeconomic, and intra-household factors (Joe et al., 2017). Due to the presence of such selectivity bias, gender differentials in use of DFH are likely to be underestimated. To address this concern, we used two-stage sample selection model (Heckman, 1979; Van de Ven and Van Praag, 1981; Vella, 1998). In the first stage, we predict the probability of becoming sick and subsequently getting hospitalized by including factors affecting occurrence of disease - age, age square, household members using flush latrine, household members drinking piped water, household garbage disposed properly, and household using non-smoke fuel for cooking; and factors affecting hospitalization/treatment seeking - biological child of the household head (only for young and adult), household head (only for adult and older adult), number of household members, number of members younger than five years, and number of members older than 60 or more as excluded variables (Asfaw et al., 2010; Joe et al., 2017). The predicted probabilities were then used to estimate the Inverse Mills' Ratio. The first stage analysis was carried out on whole sample of hospitalized cases (n = 156,723 young, n = 332,669 adult, and n = 45,909 older adult) to predict the probability of getting sick and being hospitalized from amongst all household members. The second stage model includes the Inverse Mills' Ratio to account for sample selection bias (Heckman, 1979), and uses the final analytic samples (n = 27,323 hospitalization cases of MCHE and n = 11,908 hospitalization cases of SCHE) (See Appendix Table 2 for first stage result).

Finally, in order to test whether health insurance cover affects the gender bias in household hospitalization financing strategy across age groups and level of catastrophic hospitalization expenditures, we have conducted two additional sub-sample analyses – 1) indexed hospitalization covered with health insurance and 2) indexed hospitalization not covered with health insurance.

## 3 Results

Across age groups, a higher percentage of hospitalization was distressed financed in households that incurred SCHE compared with households that incurred MCHE. Among the young, only one-third (31–33%) of hospitalized cases were female (Table 1). Among older adults, 37–42% of hospitalized were females. In comparison, about 47–48% of hospitalized cases among adults were females. Females constitute lower proportions of total hospitalization cases from households that incurred SCHE compared with households that incurred MCHE.

**Table 1:** Characteristics of hospitalized cases@ from household incurred catastrophic hospitalization expenditure in last 365 days, India 2017-18.

	Young				Adult				Older adult			
	MCHE		SCHE		MCHE		SCHE		MCHE		SCHE	
	Mean	SE	Mean	SE	Mean	SE	Mean	SE	Mean	SE	Mean	SE
Outcome												
Used distressed financing for hospitalization (%)	24.9	1.5	32.8	2.8	25.1	0.7	32.0	1.3	23.7	1.0	28.9	1.4
Exposure												
Female (%)	33.1	1.5	31.2	2.9	48.4	0.8	46.8	1.3	41.7	1.2	37.3	1.6
Hospitalization & treatment characteristics												
Number of hospitalizations in household (mean)	2.2	0.1	2.5	0.1	1.7	0.0	2.0	0.0	2.0	0.0	2.3	0.1
Nature of ailment												
Cardio-vascular diseases (%)	4.6	0.8	8.1	1.8	9.8	0.4	10.3	0.6	19.1	0.9	22.6	1.4
Cancers (%)	0.6	0.1	1.4	0.4	4.3	0.4	6.7	0.8	7.0	0.8	11.1	1.4
Musculoskeletal diseases (%)	1.1	0.2	1.3	0.3	5.6	0.5	7.5	1.1	6.4	0.5	6.1	0.7
Communicable diseases (%)	51.0	1.6	38.5	2.6	24.9	0.7	17.9	1.1	15.4	0.9	10.6	0.9
Other disease (%)												
Hospitalized at private health care facility (%)	78.6	1.1	84.3	1.5	77.2	0.6	79.6	0.9	73.5	1.0	78.7	1.5
Duration of hospitalization (days - mean)	7.8	0.3	10.3	0.6	9.0	0.3	12.0	0.6	8.9	0.3	10.5	1.5
Out-of-Pocket hospitalization expenditure in last 365 days (INR₹ - mean)	27820	1229	49482	2896	39368	1085	64344	2107	47069	1537	73700	2870
Share of Out-of-Pocket hospitalization expenditure to total household expenditure (%)	15.2	0.4	22.9	0.9	20.4	0.4	29.7	0.7	20.0	0.3	27.3	0.6
Economic characteristics												
Non-medical monthly per capita consumption expenditure (INR₹ - mean)	2056	43	1899	64	2363	22	2227	34	2836	41	2879	57
Health insured (%)	16.7	1.2	19.1	2.6	20.6	0.6	19.2	0.9	20.4	0.9	18.5	1.2
Household head regular salary/wage employee (%)	16.0	1.1	14.7	2.3	14.4	0.5	12.7	0.7	NA		NA	
Household head currently pensioner/rentier/remittances recipients (%)	3.1	0.4	3.7	0.8	4.5	0.2	4.9	0.3	NA		NA	
Regular salary/wage employee (%)	NA		NA		8.4	0.4	7.9	0.6	0.9	0.1	1.1	0.2
Currently pensioner/rentier/remittances recipients (%)	NA		NA		1.6	0.1	1.6	0.2	22.4	0.8	22.8	1.2
Social Characteristics												
Household head secondary education completed (%)	36.0	1.7	34.1	3.0	33.1	0.7	30.6	1.1	NA		NA	
Secondary education completed (%)	NA		NA		36.2	0.8	32.8	1.2	20.5	0.8	22.6	1.2
Currently married (%)	NA		NA		73.8	0.7	73.6	1.1	60.8	1.2	63.9	1.6
Female household head (%)	9.1	0.9	12.2	1.9	11.3	0.5	12.0	0.9	NA		NA	
Scheduled Caste/Tribe (%)	20.9	1.1	20.1	1.8	24.6	0.8	25.8	1.3	17.9	1.0	16.9	1.4
Hindu (%)	79.7	1.2	84.3	1.6	81.4	0.6	83.4	1.0	78.8	0.9	79.6	1.2
Rural residence (%)	72.4	1.4	79.4	1.8	68.7	0.7	72.4	1.0	62.3	1.1	64.0	1.4
Number	3484		1225		16758		7254		7081		3429	

MCHE = Share of OOPHE to THE >= 10%; SCHE = Share of OOPHE to THE >= 25%; @ Cases refers episodes of individual hospitalization in last 365 days; NA = Not applicable.

Table 2 shows bivariate association between use of DFH and independent and control variables across age groups for MCHE and SCHE. Among young and adults, a lesser percentage of female hospitalizations were distressed financed compared with male hospitalizations across catastrophic hospitalization expenditures. Moreover, gender gap in use of DFH is more visible in households that incurred SCHE relative to MCHE. The gender gap in use of DFH was statistically insignificant among older adults in both the MCHE and SCHE groups.

**Table 2:** Percentage hospitalization distressed financed by male and female from household incurred catastrophic hospitalization expenditure in last 365 days, India 2017-18.

	Young				Adult				Older adult			
	MCHE		SCHE		MCHE		SCHE		MCHE		SCHE	
	%	p-val	%	p-val	%	p-val	%	p-val	%	p-val	%	p-val
Male	26	0.01	36	0.00	26	0.00	34	0.00	23	0.62	28	0.10
Female	22		26		24		30		24		31	
Number	3484		1225		16758		7254		7081		3429	

MCHE = Share of OOPHE to THE >= 10%; SCHE = Share of OOPHE to THE >= 25%; NA = Not applicable.

Table 3 shows adjusted marginal effects from probit regression for the use of DFH among young, adults and older adults in households that incurred MCHE and SCHE without adjusting for sample selection bias. Across age groups and households that incurred MCHE and SCHE, there were no significant gender differences in the use of DFH. However, following adjustment for sample selection bias, there were clear gender differentials in use of DFH among young and older adults in households that incurred SCHE (Table 4). Among the young, the probability of use of DFH among females was 10% points less compared with males. Likewise, among the older adults, probability of use of DFH in females was 10% points less compared with males. Sample selection-adjusted results also show that across age groups, household that incurred MCHE did not have significant gender differentials in use of DFH.

**Table 3:** Marginal effect (ME) of probit regression for using distressed financing for hospitalization in the household incurred catastrophic hospitalization expenditure in last 365 days, India 2017-18.

	Young		Adult		Older adult	
	MCHE	SCHE	MCHE	SCHE	MCHE	SCHE
	ME 95%CI	ME 95%CI	ME 95%CI	ME 95%CI	ME 95%CI	ME 95%CI
Exposure						
Female (male®/female)	−0.03 (−0.08,0.02)	−0.08 (−0.16,0.00)	−0.01 (−0.03,0.02)	−0.01 (−0.05,0.03)	<0.01 (−0.04,0.03)	−0.01 (−0.06,0.04)
Hospitalization & treatment characteristics						
Number of hospitalizations in household	0.01 (−0.01,0.02)	0.01 (−0.01,0.04)	0.03* (0.02,0.04)	0.02* (0.00,0.03)	0.02* (0.00,0.03)	0.02 (0.00,0.04)
Nature of ailment						
Other disease®						
Cardio-vascular disease	−0.02 (−0.12,0.07)	−0.01 (−0.14,0.13)	−0.01 (−0.04,0.03)	−0.02 (−0.08,0.04)	−0.05* (−0.10,-0.01)	−0.09* (−0.15,-0.03)
Cancers	−0.08 (−0.29,0.13)	−0.13 (−0.37,0.12)	0.03 (−0.03,0.10)	0.07 (−0.02,0.16)	−0.11* (−0.20,-0.02)	−0.10 (−0.22,0.01)
Musculoskeletal disease	−0.01 (−0.13,0.12)	−0.03 (−0.24,0.19)	−0.05 (−0.13,0.03)	−0.09 (−0.22,0.03)	0.01 (−0.05,0.07)	0.03 (−0.06,0.12)
Communicable disease	−0.01 (−0.06,0.03)	0.01 (−0.08,0.09)	<0.01 (−0.04,0.03)	−0.02 (−0.08,0.04)	−0.04 (−0.08,0.01)	−0.01 (−0.08,0.06)
Hospitalized at private health care facility (no®/yes)	0.06 (0.00,0.11)	0.02 (−0.09,0.12)	−0.06* (−0.09,-0.02)	−0.05 (−0.11,0.01)	−0.02 (−0.06,0.03)	−0.01 (−0.07,0.06)
Duration of hospitalization (days)	<0.01 (0.00,<0.01)	<0.01 (0.00,<0.01)	<0.01 (0.00,<0.01)	<0.01 (0.00,<0.01)	<0.01 (0.00,<0.01)	<0.01 (0.00,<0.01)
Natural log of Out-of-Pocket hospitalization expenditure in last 365 days (INR₹)	0.04* (0.00,0.08)	0.04 (−0.02,0.11)	0.07* (0.05,0.09)	0.09* (0.05,0.12)	0.02 (0.00,0.04)	0.02 (−0.01,0.05)
Share of Out-of-Pocket hospitalization expenditure to total household expenditure (%)	<0.01* (0.00,0.01)	<0.01* (0.00,0.01)	<0.01 (0.00,<0.01)	<0.01 (0.00,<0.01)	<0.01* (0.00,<0.01)	<0.01* (0.00,<0.01)
Economic characteristics						
Natural log of non-medical monthly per capita consumption expenditure (INR₹)	−0.03 (−0.09,0.02)	−0.08 (−0.18,0.01)	−0.06* (−0.09,-0.03)	−0.07* (−0.11,-0.02)	−0.01 (−0.04,0.03)	<0.01 (−0.05,0.04)
Health insured (no®/yes)	0.01 (−0.06,0.07)	0.07 (−0.05,0.19)	0.01 (−0.02,0.04)	0.01 (−0.04,0.06)	0.01 (−0.03,0.06)	0.09* (0.03,0.16)
Household head regular salary/wage employee (no®/yes)	0.03 (−0.04,0.09)	0.06 (−0.06,0.18)	−0.01 (−0.05,0.02)	<0.01 (−0.07,0.06)	NA	NA
Household head currently pensioner/rentier/remittances recipients (no®/yes)	−0.12* (−0.23,-0.01)	−0.15 (−0.33,0.03)	−0.05 (−0.1,0.01)	−0.05 (−0.13,0.03)	NA	NA
Regular salary/wage employee (no®/yes)	NA	NA	−0.03 (−0.08,0.01)	−0.07 (−0.14,0.01)	0.01 (−0.13,0.16)	−0.05 (−0.21,0.11)
Currently pensioner/rentier/remittances recipients (no®/yes)	NA	NA	0.08* (0.00,0.16)	0.13* (0.01,0.25)	0.03 (−0.01,0.06)	0.07* (0.01,0.12)
Social Characteristics						
Household head secondary education completed (no®/yes)	−0.07* (−0.13,-0.02)	−0.11* (−0.20,-0.01)	−0.05* (−0.09,-0.02)	−0.04 (−0.10,0.02)	NA	NA
Secondary education completed (no®/yes)	NA	NA	−0.03 (−0.06,0.01)	−0.04 (−0.10,0.02)	−0.11* (−0.15,-0.06)	−0.17* (−0.23,-0.10)
Currently married (no®/yes)	NA	NA	−0.01 (−0.04,0.02)	<0.01 (−0.04,0.05)	−0.04* (−0.08,0.00)	−0.05* (−0.10,0.00)
Female household head (no®/yes)	0.06 (−0.01,0.14)	0.08 (−0.02,0.19)	0.07* (0.03,0.10)	0.10* (0.04,0.15)	NA	NA
Scheduled Caste/Tribe (no®/yes)	0.05* (0.00,0.10)	0.09* (0.01,0.17)	0.01 (−0.02,0.04)	0.01 (−0.04,0.05)	0.04 (−0.01,0.08)	<0.00 (−0.05,0.06)
Hindu (no®/yes)	−0.04 (−0.10,0.03)	−0.01 (−0.12,0.10)	<0.01 (−0.03,0.04)	0.01 (−0.05,0.06)	<0.01 (−0.04,0.04)	−0.01 (−0.07,0.05)
Rural residence (no®/yes)	0.01 (−0.04,0.06)	−0.01 (−0.09,0.08)	0.01 (−0.02,0.04)	−0.01 (−0.06,0.04)	0.03 (−0.01,0.07)	0.06* (0.00,0.12)
Number	3484	1225	16575	7254	7081	3429

MCHE = Share of OOPHE to THE >= 10%; SCHE = Share of OOPHE to THE >= 25%; NA = Not applicable; *Statistically significant at p<0.05; State-level fixed effects included.

**Table 4:** Marginal effect (ME) of sample selection probit regression for using distressed financing for hospitalization in the household incurred catastrophic hospitalization expenditure in last 365 days, India 2017-18.

	Young		Adult		Older adult	
	MCHE	SCHE	MCHE	SCHE	MCHE	SCHE
	ME 95%CI	ME 95%CI	ME 95%CI	ME 95%CI	ME 95%CI	ME 95%CI
Exposure						
Female (male®/female)	−0.03 (−0.08,0.02)	−0.10* (−0.19,-0.02)	<0.01 (−0.03,0.02)	−0.01 (−0.05,0.03)	−0.04 (−0.09,0.01)	−0.10* (−0.17,-0.03)
Hospitalization & treatment characteristics						
Number of hospitalizations in household	0.01 (−0.01,0.02)	0.01 (−0.01,0.04)	0.03* (0.02,0.04)	0.02* (0.00,0.03)	0.02* (0.00,0.03)	0.02* (0.00,0.04)
Nature of ailment						
Other disease®						
Cardio-vascular disease	−0.02 (−0.12,0.07)	0.01 (−0.13,0.14)	<0.01 (−0.03,0.04)	−0.01 (−0.07,0.05)	−0.06* (−0.10,-0.01)	−0.10* (−0.15,-0.04)
Cancers	−0.08 (−0.28,0.13)	−0.14 (−0.38,0.11)	0.05 (−0.02,0.11)	0.09 (0.00,0.18)	−0.11* (−0.20,-0.02)	−0.1 (−0.22,0.01)
Musculoskeletal disease	−0.01 (−0.13,0.12)	−0.03 (−0.25,0.18)	−0.05 (−0.12,0.03)	−0.09 (−0.21,0.04)	0.01 (−0.05,0.07)	0.03 (−0.06,0.12)
Communicable disease	−0.01 (−0.06,0.03)	0.01 (−0.07,0.09)	−0.01 (−0.04,0.03)	−0.02 (−0.08,0.04)	−0.04 (−0.08,0.01)	−0.01 (−0.08,0.06)
Hospitalized at private health care facility (no®/yes)	0.06 (0.00,0.11)	0.02 (−0.08,0.12)	−0.06* (−0.09,-0.02)	−0.05 (−0.11,0.01)	−0.02 (−0.06,0.03)	−0.01 (−0.08,0.06)
Duration of hospitalization (days)	<0.01 (0.00,<0.01)	<0.01 (0.00,<0.01)	<0.01 (0.00,<0.01)	<0.01 (0.00,<0.01)	<0.01 (0.00,<0.01)	<0.01 (0.00,<0.01)
Natural log of Out-of-Pocket hospitalization expenditure in last 365 days (INR₹)	0.04* (0.00,0.08)	0.04 (−0.02,0.11)	0.07* (0.05,0.09)	0.08* (0.05,0.11)	0.02 (0.00,0.04)	0.02 (0.00,0.05)
Share of Out-of-Pocket hospitalization expenditure to total household expenditure (%)	<0.01* (0.00,0.01)	<0.01* (0.00,0.01)	<0.01 (0.00,<0.01)	<0.01 (0.00,<0.01)	<0.01* (0.00,<0.01)	<0.01* (0.00,<0.01)
Economic characteristics						
Natural log of non-medical monthly per capita consumption expenditure (INR₹)	−0.03 (−0.09,0.02)	−0.06 (−0.16,0.04)	−0.03 (−0.07,0.0)	−0.03 (−0.09,0.02)	0.03 (−0.02,0.07)	0.08* (0.02,0.14)
Health insured (no®/yes)						
Household head regular salary/wage employee (no®/yes)	0.03 (−0.04,0.09)	0.06 (−0.06,0.18)	−0.01 (−0.05,0.03)	0.01 (−0.06,0.07)	NA	NA
Household head currently pensioner/rentier/remittances recipients (no®/yes)	−0.12* (−0.23,-0.01)	−0.15 (−0.33,0.03)	−0.06* (−0.11,0.00)	−0.06 (−0.14,0.02)	NA	NA
Regular salary/wage employee (no®/yes)	NA	NA	−0.04 (−0.09,0.00)	−0.08* (−0.16,-0.01)	−0.03 (−0.18,0.12)	−0.15 (−0.32,0.01)
Currently pensioner/rentier/remittances recipients (no®/yes)	NA	NA	0.11* (0.03,0.19)	0.17* (0.04,0.29)	0.02 (−0.01,0.06)	0.06* (0.01,0.11)
Social Characteristics						
Household head secondary education completed (no®/yes)	−0.07* (−0.13,-0.02)	−0.10* (−0.20,-0.01)	−0.04* (−0.08,-0.01)	−0.03 (−0.09,0.03)	NA	NA
Secondary education completed (no®/yes)	NA	NA	−0.06* (−0.10,-0.02)	−0.09* (−0.16,-0.01)	−0.13* (−0.19,-0.08)	−0.22* (−0.29,-0.15)
Currently married (no®/yes)	NA	NA	<0.01 (−0.03,0.02)	0.01 (−0.04,0.05)	−0.07* (−0.12,-0.03)	−0.13* (−0.19,-0.06)
Female household head (no®/yes)	0.06 (−0.01,0.14)	0.08 (−0.02,0.18)	0.08* (0.04,0.11)	0.11* (0.05,0.17)	NA	NA
Scheduled Caste/Tribe (no®/yes)	0.05* (0.00,0.10)	0.08* (0.00,0.17)	0.01 (−0.02,0.04)	<0.01 (−0.04,0.05)	0.04 (−0.01,0.08)	<0.01 (−0.06,0.05)
Hindu (no®/yes)	−0.04 (−0.10,0.03)	<0.01 (−0.11,0.11)	<0.01 (−0.03,0.03)	<0.01 (−0.06,0.06)	−0.01 (−0.05,0.03)	−0.03 (−0.09,0.03)
Rural residence (no®/yes)	0.01 (−0.04,0.06)	−0.01 (−0.09,0.08)	0.01 (−0.02,0.04)	−0.01 (−0.06,0.04)	0.02 (−0.01,0.06)	0.05 (−0.01,0.10)
Inverse Mill's Ratio	−0.02 (−0.49,0.46)	0.37 (−0.36,1.09)	0.56* (0.18,0.94)	0.65* (0.09,1.21)	0.67 (−0.03,1.36)	1.45* (0.67,2.24)
Selected	3484	1225	16575	7254	7081	3429
Non-selected	153239	155498	316094	325415	38828	42478

MCHE = Share of OOPHE to THE >= 10%; SCHE = Share of OOPHE to THE >= 25%; NA = Not applicable; *Statistically significant at p<0.05; State-level fixed effects included.

To examine whether gender differentials in use of DFH vary by whether index case is covered for health expenditure or not, we estimated marginal effects separately for the two levels of catastrophic hospitalization expenditures (Table 5). In households that incurred SCHE, probability of use of DFH of female older adults was lower compared with male older adults, irrespective of whether they were health insured or not. Similarly, in households that incurred SCHE and were not covered by health insurance, probability of use of DFH of young females was lower compared with young males. In comparison, in households that incurred SCHE and were covered by health insurance, young female hospitalizations were no less likely than young male hospitalizations to get distressed financed.

**Table 5:** Sample selection adjusted probit regression marginal effect (ME) for female for using distressed financing for hospitalization in the household incurred catastrophic hospitalization expenditure in 365 days, India 2017-18.

	MCHE		SCHE	
	ME 95%CI	Number	ME 95%CI	Number
Health insured				
Young (male®/female)	0.05 (−0.07,0.16)	536	0.08 (−0.10,0.27)	205
Adult (male®/female)	<0.01 (−0.05,0.05)	3286	<0.01 (−0.09,0.08)	1392
Older adult (male®/female)	−0.07 (−0.17,0.03)	1344	−0.17* (−0.30,-0.03)	608
Not health insured				
Young (male®/female)	−0.03 (−0.09,0.02)	2948	−0.13* (−0.22,-0.05)	1020
Adult (male®/female)	<0.01 (−0.03,0.02)	13472	−0.02 (−0.06,0.03)	5862
Older adult (male®/female)	−0.03† (−0.09,0.02)	5737	−0.07† (−0.14,0.00)	2820

Result is adjusted for all corresponding variables included in Table 4; MCHE = Share of OOPHE to THE ≥ 10%.

SCHE = Share of OOPHE to THE ≥ 25%; *Statistically significant at p < 0.05, †Statistically significant at p < 0.10.

State-level fixed effects included.

## 4 Discussion

This study establishes clear gender differences in the use of distressed financing - borrowing, selling of assets, and contribution from relatives or friends - for hospitalization financing for young and for older adults in India in households that incurred SCHE. These findings are consistent with those seen in prior research from India among adults (Moradhvaj and Saikia, 2019), children (Asfaw et al., 2010), and older adults (Joe et al., 2017). However, this research extends prior work by demonstrating that these male-biased hospitalization financing strategies are specific to catastrophic expenditures. Our findings support the growing body of evidence that gender discrimination in health expenditures and financing strategies is not limited to young individuals in India but is an issue affecting older populations as well.

These findings suggest that gender differentials in the use of DFH are likely affected by the social and economic roles and value that a female is perceived to offer the family. Gender differentials are not seen for those of working and childbearing age, possibly because women's value as child bearer and care provider for the family offers value. However, older adult females, perceived to offer lesser social and economic value for family (Kalavar and Jamuna, 2011), may be deprioritized for the use of DFH. Findings from this research correspond with prior studies demonstrating lower likelihood of both necessary hospitalizations and the use of DFH for adult women relative to adult men (Moradhvaj and Saikia, 2019). This study further extends these findings by indicating that differences appears to be specific to older adult women rather than women of childbearing and working age. Joe et al. (2017) have also shown that DFH was higher among older males compared to older females. Study findings also show gender differentials in DFH among young individuals, a finding that corresponds with prior research demonstrating differential health care seeking by gender for young children (Mathew, 2012; Vilms et al., 2017; Willis et al., 2009) as well as early adolescents 10–14 years (Gupta et al., 2016). In tandem, our results highlight that gender bias, often perceived and studied among young, is in fact equally experienced among older adults. Gender differentials in the use of DFH are also likely affected, although to a smaller extent, by greater exposure of adult men to lifestyle risk factors such as smoking, drinking, stress, substance abuse, etc. as identified in other studies (Sarma et al., 2019; Thakur et al., 2019). Higher exposure to life style risk factors among men might lead to high intensity care requiring greater expenditure.

As the gender bias against young and older adult females in the use of DFH that we find adjusts for economic characteristics, we are not simply picking up greater gender bias for economic disadvantaged compared to economic well-off populations. Rather, we are likely seeing the persistence of gender bias and gender discrimination in India regardless of economic status, and action on this bias is more clearly illuminated in contexts where choice in investment is required. When that choice is required, financial investment is more likely to be made for males rather than females. The combination of gender bias and perceptions of lesser economic capital gain for young and older adults renders girls and older women less valuable, and thus less worthy of family investment in health. The Government of India, in its National Health Policy 2017, has envisaged to reduce OOPHE due to health care costs and achieve reduction in proportion of households experiencing catastrophic health expenditure and consequent impoverishment (MOHFW, 2017). The policy if implemented in totality is likely to reduce the incidence of CHE in general and gender discrimination in DFH in particular among young and older adult.

Gender differentials in the use of DFH does not occur among young and older adults until share of OOPHE becomes equal or more than 25% of THE. We tested additional cut points of OOPHE at 15% and 20% of THE, and there was not a significant gender bias in any assessed age groups (results not shown). We also conducted a sensitivity analysis in which we categorized states in two groups - EAG,^1^ where public health provision or infrastructure are poor and Non-EAG, where the public health provision or infrastructure are relatively better (NRHM, 2005). We conducted this sensitivity analysis to test whether better provision of public health care reduces the gender gap in use of DFH across age groups and genders (See Appendix Table 3). Result shows that use of DFH was 11% (MCHE) and 21% (SCHE) points less among young females from EAG states compared with young males. In contrast, there was no difference in the use of DFH between young males and females in Non-EAG states. Similar differences are found among older adults; but marginal effects were not significant. These findings reinforce the need for either free public health provisions or financial protection to reduce OOPHE to ensure that neither access nor financial constraints affect health care utilization or its financing decision-making for female household members. The recently launched health assurance scheme, Ayushman Bharat Pradhan Mantri Jan Arogya Yojana (ABPMJAY), covering INR₹500,000 (a little over 7000 USD) per family per year from the bottom 40% of the Indian population is significant step in this direction (National Health Authority, 2018). Young girls and female older adults in households incurring exorbitant amount of health expenditure are likely to benefit more from this scheme.

The finding that health expenditure cover, in the form of health insurance or health assurance schemes, is not likely to protect the female older adults from household discrimination in hospitalization financing strategy for SCHE is a matter of great concern, and requires immediate attention from policy makers and program managers. Although the ABPMJAY may financially protect the households from health care spending on its members, it may not protect the older females from household discrimination in hospitalization financing strategies. Such a finding calls for greater focus on enhancing the value of female older adults in the households. Special public health care provisions targeted at older adult females along with health insurance or assurance schemes may also help.

## 5 Limitations

This study should be interpreted in light of its limitations. NSSO surveys, which are cross-sectional surveys, do not collect information on whether a person requiring hospitalization was actually hospitalized, which limits the ability to comprehensively address selectivity bias. While outpatient expenditure, in NSSO, is collected only in last 15 days, the inpatient expenditure is collected for last 365 days. Hence, we could not include the outpatient expenditure in total household expenditure. This is less of a problem as Deaton and Zaidi (2002) have argued that consumption aggregation may not include health expenses if its income elasticity is less than the measurement error it creates in aggregate household consumption. Since the income elasticity for health expenses is 0.47 (less than 1 – relatively inelastic) in India (Rahman, 2008), the measurement errors are likely to be huge if we convert 15 days outpatient expenditure to 365 days expenses by multiplying with 24.3. Moreover, since we are calculating out-of-pocket hospitalization expenditure and catastrophic hospitalization expenditure, the exclusion of outpatient expenditure in THE is not likely to affect our results. Additionally, responses may have been influenced by recall and self-report bias, and we cannot infer causality from these cross-sectional data.

## 6 Conclusions

This study documents significant gender bias in the use of distressed financing - borrowing, selling of assets, and contribution from relatives or friends – in cases of hospitalization in India for young and older adults, with distressed financing less likely to be used for females than for males. This is suggestive of gender bias in health care financing based on greater perceived value of males relative to females in the family, particularly when the females are young or older adults. Increased valuation of girls and elderly women throughout their life, as well as improved responses from the health system to support girl children and older women in economically deprived circumstances, are needed to systematically support the currently aspirational goal of universal health care for all. For future studies, our results show the persistence of son preference in India in ways that are not necessarily captured by sex ratios and highlight the need for broader social change to value the girl child.

## Credit author statement

Kaushalendra Kumar (KK) and Abhishek Singh (AS) conceptualized the analysis; KK conducted data analysis; AS and Lotus McDougal (LM) supervised the data analysis; KK, AS and Anita Raj (AR) wrote the first draft; K S James (KSJ), LM, and AR reviewed the first draft and edited the paper.

## Declaration of competing interest

None.

## Figures and Tables

**Appendix Table 1:** Classification of diseases

**Cardio-vascular disease** – Hypertension, Heart disease: Chest pain, breathlessness.
**Cancers** – Known or suspected by a physician and occurrence of any growing painless lump in the body.
**Musculoskeletal disease** – Joint or bone disease/pain or swelling in any of the joints, or swelling or pus from the bones, Back or body aches.
**Communicable diseases** – Diphtheria, Whooping Cough, Malaria, Typhoid, Tuberculosis, Filariasis, Tetanus, HIV/AIDS, STI, Jaundice, Diarrhoea, Dysentery, Worm Infestation, Fever with rash/Eruptive lesions.
**Other disease** – Anaemia (any cause), Bleeding disorders, Diabetes, Under-nutrition, Goitre and other diseases of the thyroid, Others (including obesity), Mental retardation, Mental disorders, Headache, Seizures or known epilepsy, Weakness in limb muscles and difficulty in movements, Stroke/hemiplegia/sudden onset weakness or loss of speech in half of body, Others including memory loss, confusion, Cataract, Glaucoma, Decreased vision (chronic) NOT including where decreased vision is corrected with glasses, Others (including disorders of eye movements– strabismus, nystagmus, ptosis and adnexa), Decreased hearing or loss of hearing, Bronchial asthma/recurrent episode of wheezing and breathlessness with or without cough over long periods or known asthma), Diseases of mouth/teeth/gums, Pain in abdomen: Gastric and peptic ulcers/acid reflux/acute abdomen, Lump or fluid in abdomen or scrotum, Gastrointestinal bleeding, Any difficulty or abnormality in urination, Change/irregularity in menstrual cycle or excessive bleeding/pain during menstruation and any other gynaecological and andrological disorders incl. male/female infertility, Illness in the new-born/sick new-born, Accidental injury, road traffic accidents and falls, Accidental drowning and submersion, Burns and corrosions, Poisoning, Intentional self-harm, Assault, Contact with venomous/harm-causing animals and plants, Symptom not fitting into any of above categories, Could not even state the main symptom.

**Appendix Table 2:** First stage result from probit regression for becoming sick and hospitalized, India 2017-18

	Young	Adult	Older adult
Age	−0.10* (−0.12,-0.09)	0.01* (0.00,0.02)	0.06* (0.01,0.10)
Age square	<0.01* (0.00,0.01)	<0.01 (0.00,<0.01)	<0.01 (0.00,<0.01)
Biological child of the household head (no®/yes)	0.02 (−0.02,0.06)	−0.12* (−0.16,-0.08)	NA
Household head (no®/yes)	NA	−0.07* (−0.11,-0.04)	−0.08* (−0.14,-0.02)
Number of household member	−0.03* (−0.04,-0.01)	−0.01* (−0.01,0.00)	−0.01 (−0.00.02,0)
Number of member younger than five years	−0.06* (−0.09,-0.03)	−0.01 (−0.03,0.01)	0.01 (−0.04,0.06)
Number of member 60 years and above	0.04* (0.01,0.07)	−0.02* (−0.04,0.00)	0.07* (0.02,0.12)
Household members using flush latrine (no®/yes)	<0.01 (−0.04,0.04)	<0.01 (−0.02,0.03)	0.01 (−0.04,0.07)
Household members drinking piped water (no®/yes)	0.03 (−0.02,0.08)	<0.01 (−0.03,0.02)	−0.03 (−0.08,0.02)
Household garbage disposed properly (no®/yes)	−0.06* (−0.11,-0.01)	0.01 (−0.02,0.04)	0.01 (−0.05,0.06)
Household using non-smoke fuel for cooking (no®/yes)	0.02 (−0.02,0.06)	−0.03* (−0.06,0.00)	<0.01 (−0.06,0.05)

**Result is controlled for** - age, age square, household members using flush latrine, household members drinking piped water, household garbage disposed properly, and household using non-smoke fuel for cooking; and factors affecting hospitalization/treatment seeking - biological child of the household head (only for young and adult), household head (only for adult and older adult), and number of household members, female, monthly per capita non-medical consumption expenditure, Scheduled Caste/Tribe, Hindu, rural residence, health expenditure cover; household head secondary education completed, household head regular salary/wage employee, household head currently pensioner/rentier/remittances recipients, female household head (only for young and adult); secondary education completed, regular salary/wage employee, pensioner/rentier/remittances recipients, household head, currently married (only for adult and older adult).

NA = Not applicable; 95% Confidence Interval (CI) is in parenthesis; State-level fixed effects included; *Statistically significant at p < 0.05.

**Appendix Table 3:** Sample selection adjusted probit regression marginal effect (ME) for female for using distressed financing for hospitalization in the household incurred catastrophic hospitalization expenditure in 365 days, EAG States and Non-EAG States-2017-18

	MCHE	SCHE
	ME 95%CI	ME 95%CI
Young		
Male from Non-EAG States®		
Male from EAG States	−0.02 (−0.08,0.05)	−0.04 (−0.16,0.08)
Female from Non-EAG States	0.01 (−0.05,0.07)	−0.02 (−0.13,0.09)
Female from EAG States	−0.11* (−0.19,-0.04)	−0.21* (−0.33,-0.09)
Adult		
Male from Non-EAG States®		
Male from EAG States	−0.03 (−0.07,0.02)	0.01 (−0.06,0.08)
Female from Non-EAG States	−0.01 (−0.05,0.02)	−0.03 (−0.08,0.03)
Female from EAG States	−0.04* (−0.08,0.00)	−0.02 (−0.08,0.04)
Older adult		
Male from Non-EAG States®		
Male from EAG States	−0.05* (−0.1,0.00)	−0.06 (−0.13,0.00)
Female from Non-EAG States	−0.02 (−0.06,0.03)	−0.02 (−0.08,0.05)
Female from EAG States	−0.04 (−0.11,0.02)	−0.06 (−0.17,0.04)

Results are adjusted for all corresponding variables included in Table 4; MCHE = Share of OOPHE to THE ≥ 10%.

SCHE = Share of OOPHE to THE ≥ 25%; *Statistically significant at p < 0.05; EAG states include – Bihar, Uttar Pradesh, Rajasthan, Madhya Pradesh, Uttarakhand, Jharkhand, Odisha, Chhattisgarh.

## Footnotes

1 Empowered Action Group (EAG) states are – Bihar, Uttar Pradesh, Rajasthan, Madhya Pradesh, Uttarakhand, Jharkhand, Odisha, Chhattisgarh.
